# Beyond neurons: astrocytic ensembles stabilize memories after recall

**DOI:** 10.1038/s41392-025-02537-9

**Published:** 2026-01-20

**Authors:** May Bakr, Mohamed Salama

**Affiliations:** Institute of Global Health and Human Ecology, School of Sciences and Engineering, American University in Cairo, Cairo, Egypt

**Keywords:** Cellular neuroscience, Cell biology

In a recent study published in *Nature*, Dewa et al. showed that emotionally salient experiences “biologically tag” small ensembles of astrocytes, creating multiday traces that can be reactivated during recall.^1^ This mechanism reveals a previously unrecognized astrocytic contribution to the stabilization of recalled memories and expands the traditional neuron-centric model of memory storage, offering insights that may inform future strategies for modulating memory in cognitive and neuropsychiatric disorders.^1^

Memory stability has traditionally been attributed to neuronal engrams, the specific populations of neurons that undergo lasting molecular and synaptic modifications during learning and are reactivated during recall.^2^ Although engram reactivation is necessary for memory expression, it does not fully explain why some memories endure, whereas others fade. Astrocytes, star-shaped glial cells that contact neurons and detect neuromodulatory signals, have therefore emerged as potential modulators of memory-related circuit function,^3^ yet their role in long-term memory stability has remained unclear.^4^

Earlier this year, Williamson et al. reported that learning induces small subsets of hippocampal astrocytes to express the activity gene Fos and that reactivating these astrocyte ensembles can trigger memory recall.^5^ However, their fear conditioning paradigm was conducted without prior habituation, allowing contextual novelty to drive much of the astrocytic Fos induction during learning. In contrast, Dewa et al. conducted fear conditioning only after three days of habituation, minimizing novelty-related activation and revealing that astrocytes are recruited selectively during recall rather than during learning.^1^ This methodological difference explains the contrasting patterns of astrocytic activation in the two studies and helps reconcile their findings. On this basis, the study by Dewa et al. is the first to demonstrate that astrocytes can serve as persistent memory traces spanning days, actively stabilizing a memory during the critical period after recall.^1^

To explore astrocytes’ involvement in memory, the researchers developed a genetic tagging system that allowed precise tracking of astrocyte activation across the whole brain.^1^ Mice were engineered so that any astrocyte expressing Fos during a defined time window would become fluorescently labeled. Using this system, the authors mapped astrocyte activation during memory formation and recall. Mice were then trained in a fear conditioning task and tested days later for recall.

**Fig. 1:**
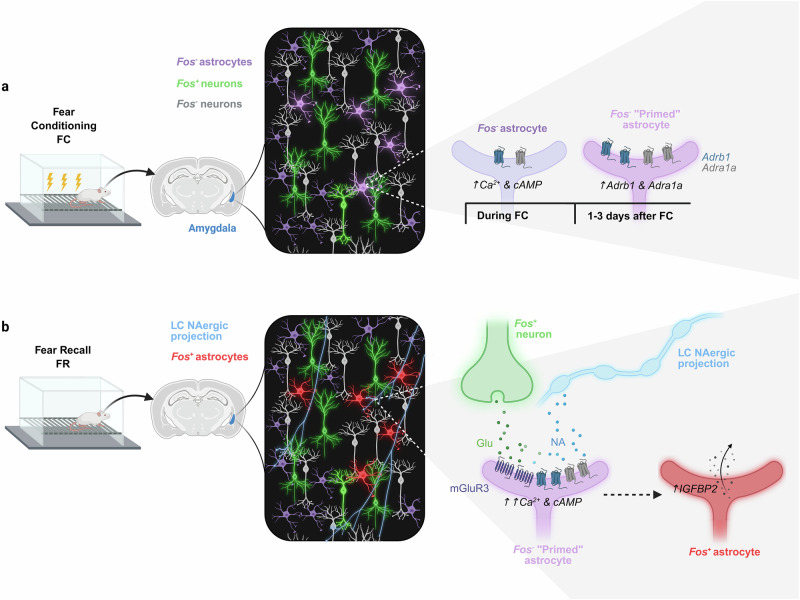
Fear learning primes lateral/basolateral amygdala astrocytes for recall-dependent memory stabilization. **a** During fear conditioning (FC), neurons in the lateral/basolateral amygdala (LA/B) exhibit robust Fos expression, whereas astrocytes remain Fos⁻ despite showing rapid Ca²⁺ and cAMP increases (left). Over the following 1–3 days, a subset of Fos⁻ astrocytes undergo learning-dependent transcriptional priming, characterized by upregulation of the adrenergic receptors Adrb1 (β₁) and Adra1a (α₁A) (right). **b** During fear recall (FR), reactivation of the neuronal engram (glutamatergic input) coincides with prolonged noradrenergic signaling from the locus coeruleus. Only primed LA/B astrocytes respond to this convergence with increased Ca²⁺ event frequency and a progressive cAMP rise, leading to Fos induction. These recall-activated astrocytes upregulate Igfbp2, supporting stabilization of the reactivated neuronal engram. Created with Biorender.com

Strikingly, astrocytes showed minimal activation during initial learning but were strongly activated during recall. Recall engaged a discrete ensemble of astrocytes, behaviorally relevant astrocyte ensembles (BAEs), particularly within the amygdala, a central hub of fear memory processing (Fig. 1a). Neurons, in contrast, displayed Fos expression during both learning and recall.^1^ Although astrocytes exhibited rapid Ca²⁺ and cAMP increases during fear conditioning, these signals were not accompanied by Fos expression (Fig. 1a). The recall-specific activation of astrocytes suggested that these cells were primed during learning to respond only upon future reactivation of the memory. Importantly, their perturbation did not alter initial recall, indicating that astrocytes do not encode memory content but instead regulate the post-recall stabilization window.^1^

Dewa et al. uncovered a two-step mechanism underlying this selective re-engagement. The initial fear experience triggered slow transcriptional changes in a subset of astrocytes. Single-cell RNA sequencing revealed that these astrocytes progressively upregulated α- and β-adrenergic receptors, Adrb1 (β₁) and Adra1a (α₁A), over 1–3 days following conditioning^1^ (Fig. 1a). This noradrenaline (NA)-associated priming state increased the astrocytes’ sensitivity to future noradrenergic signals. This finding aligns with earlier evidence that noradrenaline can prime astrocytes to detect local neural activity.^3^

During recall, two signals converged: the local neuronal engram was reactivated, and noradrenaline was released from locus coeruleus (LC) projections. Only astrocytes previously primed during learning responded robustly to this coincidence of signals (Fig. 1b). The convergence of glutamatergic input from reactivated engram neurons and prolonged noradrenergic inputs drove a progressive cAMP rise and increased Ca²⁺ event frequency during recall, leading to Fos induction. Once activated, the astrocytes upregulated IGFBP2^1^ (Fig. 1b), a plasticity-associated factor previously shown to be enriched in peri-engram astrocytes and required for fear memory formation,^4^ supporting their role in stabilizing the reactivated neuronal engram.

Causal manipulations provided direct support for this mechanism. Silencing the recall-activated astrocyte ensemble or suppressing adrenergic receptor signaling in the amygdala impaired memory stabilization across repeated recalls. Mice failed to show the expected fear response, demonstrating that both astrocytic activity and noradrenergic input are required for reconsolidation. Conversely, overexpression of the β₁-adrenergic receptor in astrocytes exaggerated stabilization of the initial fear memory. Treated mice behaved as if a weak fear memory were much stronger, and even generalized their fear to unfamiliar contexts, leading to overgeneralization and loss of memory precision.^1^ These findings show that the magnitude of astrocytic engagement directly shapes the strength and specificity of engram reactivation, indicating that memory retrievability is not fixed during learning but can be amplified after recall.

Collectively, these results position astrocytes as key regulators of memory persistence. By embedding a multiday priming mechanism within emotionally salient experiences and re-engaging during recall, astrocytes provide a stabilizing influence that protects important memories and shapes their long-term expression.^1^ As such, this work adds a new cellular dimension to memory biology and offers a foundation for tuning memory stability with cell-type and temporal specificity.

Looking ahead, future studies should confirm whether similar multiday astrocytic traces operate in other memory domains across different behavioral paradigms. Beyond amygdala-dependent fear learning, long-term memory relies on diverse systems, including hippocampus-dependent episodic and spatial memory and prefrontal cortex-mediated working and associative memory.^2^ Whether astrocytic tagging or priming contributes to stabilization in these regions remains an open question. Such investigations could determine whether astrocytes provide a general mechanism for memory stabilization or a circuit-specific process tuned to particular forms of learning.

At the translational level, these findings extend relevance beyond post-traumatic stress disorders (PTSD) and other disorders marked by maladaptive memory persistence. By grounding memory stabilization in an astrocyte-dependent biological mechanism, this work may eventually refine cognitive assessments and illuminate memory-related vulnerabilities in neurodegenerative diseases, where astrocytes are already implicated. Conceptually, recognizing astrocytes as active determinants of memory stability could also reshape how future neurotechnologies, including brain-computer interfaces, approach circuit modulation, shifting attention from neuron-centric paradigms toward glia-inclusive strategies.

Finally, a compelling question is whether variability in astrocytic priming efficiency could underlie individual differences in memory precision and performance.
